# Epidemiological profile of octogenarian patients admitted to our intensive care unit (ICU) because of an acute coronary syndrome (ACS). is there an adequate resources management?

**DOI:** 10.1186/2197-425X-3-S1-A756

**Published:** 2015-10-01

**Authors:** A Puerto-Morlán, MA Estecha-Foncea, A Fernandez Poncel, P Nuevo-Ortega, L Ruiz-Del-Fresno, C Reina-Artacho

**Affiliations:** Intensive Care Unit, Hospital Universitario Virgen de la Victoria, Malaga, Spain

## Introduction

During the last decades is becoming more frequent to admit patients older than 80 in the Intensive Care Unit (ICU). One of the most common reasons for admission of these patients is Acute Coronary Syndrome (ACS). We decide to study specifically this population, which has distinctive features but has not been fully studied because its advanced age has been an exclusion criteria in most clinical trials.

## Objectives

To describe epidemiological features of patients older than 80, admitted in a our ICU because of ACS. We analyzed mortality and functional capacity at six month time after the event.

## Methods

We carried out an observational study examining clinical records of all patients admitted in our Unit between June 1, 2013 and June 30, 2014, aged 80 or older. We collect information about basal physical and functional state, type of ACS, treatment applied and evolution. Patients or their contact relatives, were interviewed again 6 months after admission to know their vital and functional state, that was evaluated with Barthel Index (BI).

## Results

Fifty two patients were studied, 80,8% (42 patients) were admitted because of ST Elevation ACS (STEACS), and the rest Non STE ACS (NSTE AVS). In STEACS patients reperfusion strategy was Primary Percutaneous Coronary Interventionism (PPCI) in 78,5% of cases, fibrinolysis 21,4%. In patients with NSTEACS 80% were treated with PCI in 80%, and surgically 10%. In STEACS total delay between symptoms onset and PPCI was 4.6 h +/- 3.97 h; in contrast with NSTEACS where this delay was 72 h +/-53 h (mean +/- SD). Global mortality rate was 21.5% (11 patients) in ICU, and 34.6% (18) at 6 months, among STEACS patients it was 40.4% (17) and in NSTEACS patients 10% (1).

Regarding place of death, 61.1% die in ICU (11 out of 18 patients), 16.6% (3) in hospital out of ICU, and 22.2% (4) after discharge to home. Regarding 34 survivors, functional state at six month could be recovered in 28, and 50% of them had a BI score < 90 points (moderate, severe or total dependency), and 35.7% less than 60 BI points indicating severe or total dependency 6 months after the admission.

## Conclusions

Patients older than 80, admitted in ICU because of STEACS have a high mortality rate, higher than expected from previous literature: in GRACE registry ([1]), global mortality was 9.3% for patients between 75 and 85 years old and 18.4% for those older than 85. In addition, a high percentage of survivors remain with at least moderate dependency. And this in spite of an optimal treatment according to current clinical guidelines. It is necessary to design studies in this subgroup of patients, oriented not only to mortality but also to quality of life; to better identify in what patients this treatments are cost-efficient.
